# Routine follow-up transjugular liver biopsy in Fontan patients: technical considerations and safety of an initial case series and literature review

**DOI:** 10.3389/fped.2023.1204545

**Published:** 2023-11-21

**Authors:** Guido Mandilaras, Zora Meyer, Richard Mühlberg, Annabell Braun, Nikolaus A. Haas, Andre Jakob, Robert Dalla Pozza, Moritz Wildgruber, Marcus Fischer

**Affiliations:** ^1^ Department of Pediatric Cardiology and Pediatric Intensive Care, University Hospital of Munich, Ludwig-Maximilian-University Munich, Munich, Germany; ^2^ Department of Interventional Radiology, University Hospital of Munich, Ludwig-Maximilian-University Munich, Munich, Germany

**Keywords:** Fontan-associated liver disease, FALD, transjugular liver biopsy, congenital heart disease, pediatric cardiology

## Abstract

**Introduction:**

Patients with Fontan palliation are susceptible to congestive hepatopathy and Fontan-associated liver disease (FALD) because of hemodynamic changes. The staging of liver fibrosis involves various methods, including invasive biopsy. Transjugular liver biopsy (TJLB) offers a less invasive alternative, enhancing liver disease surveillance in routine cardiac catheterization. We detail the technical aspects, share initial outcomes, and discuss existing literature.

**Methods/results:**

During routine follow-up cardiac catheterization indicated by hemodynamic or clinical alterations, four patients aged between 16 and 26 years with univentricular Fontan circulation and three patients with biventricular circulation underwent TJLB during routine surveillance catheterization. The examinations were performed under conscious sedation and local anesthesia without general anesthesia. Jugular access was obtained at the site of liver localization, and a 5 F multipurpose catheter was inserted into the liver veins. After hand angiography to delineate the local hepatic venous anatomy, an exchange wire was used to place the bioptome, and three consecutive biopsies were performed. There were no complications, especially perforation or bleeding. The technical success rate was 100%, with all obtained samples appropriate for histopathological diagnostics. The total additional procedure time was less than 20 min.

**Conclusion:**

TJLB is an attractive alternative method for obtaining liver specimens in the scope of FALD care. We believe that it should be performed during routine hemodynamic evaluations in Fontan patients and can be performed safely with very low additional time expenditure. As the biopsy site is intravascular, the risk of external bleeding or hematoma is significantly reduced despite the high intrahepatic pressures and the usually impaired coagulation profile in these patients. Based on our initial experience and the lower complication rates compared with other techniques, TJLB should be considered a standard approach in these patients and used more often during the long-term follow-up of Fontan patients. It can be performed in the same setting whenever a hemodynamic assessment of patients with congenital heart defects is required.

## 1. Introduction

Patients with Fontan palliation are at risk of developing congestive hepatopathy and Fontan-associated liver disease (FALD), which is one of the severe long-term complications for this patient cohort. Hemodynamic changes, such as low cardiac output and chronic elevation of central venous pressure, contribute to the development of FALD. Chronic FALD may result in hepatic cirrhosis and hepatic cellular cancer, with possibly lethal consequences (1). The staging of liver fibrosis in FALD involves biochemical/hematological parameters, non-invasive fibrosis scores, sonographic liver elastography, and liver histology, in some cases.

Most centers caring for Fontan patients evaluate liver function using ultrasound-guided liver elastography, sometimes employing magnetic resonance elastography (MRE), and liver-specific laboratory parameters at regular intervals as part of their surveillance programs (2). When FALD progresses to severe hepatic congestion with progressive hepatic alterations, a liver biopsy is the gold standard diagnostic procedure. However, it is also highly invasive to estimate the severity of liver disease through a histopathological diagnosis (3).

Due to contraindications for performing a percutaneous liver biopsy (PLB), such as coagulopathy or ascites because of liver failure, transjugular liver biopsy (TJLB) has been gaining ground as an alternative method among adult patients for liver disease surveillance (4). In Fontan patients, this method may play an important role in the long-term assessment of liver pathology. Fontan patients often require a hemodynamic assessment because of clinical or hemodynamic alterations in the long-term follow-up (i.e., protein-losing enteropathy, reduced exercise performance, and increasing cyanosis). Whenever catheter investigations are planned, TJLB can be added to the procedure with minimal effort and performed safely by cardiac interventionalists without requiring major additional time. Therefore, we introduced TJLB in our routine cardiac catheter program. This article reports the technical aspects of this procedure, our initial results, and the potential benefits for this patient cohort. In addition, a review of the literature is provided.

## 2. Patients and methods

Since 2020, patients with biventricular circulation or especially those with univentricular heart diseases who present with clinical signs of right heart failure, elevated central venous pressure, high estimated pulmonary vascular resistance in the Fontan circulation, and signs of hepatopathy undergo TJLB during routine hemodynamic surveillance catheterization in our department (Division of Pediatric Cardiology and Pediatric Intensive Care of the University Hospital of Munich).

We present the clinical course and technical aspects of our initial four patients, aged between 16 and 26 years, with failing Fontan circulation and FALD and three patients with biventricular congenital heart defects, aged between 12 and 55 years, who underwent cardiac catheterizations and TJLB during the same procedure (see Table 1). As a standard of care in our institution, the examinations were performed under conscious sedation and local anesthesia, without general anesthesia.

**Table 1:** Patients’ characteristics and history.

Fontan circulations	Case A	Case B	Case C	Case D
Sex	Female	Male	Male	Male
Age (years)	29	16	16	17
Primary diagnosis	HLHS with aortic and mitral valve stenosis	HLHS with aortic and mitral valve stenosis	DILV with l-transposition of the great arteries, pulmonary stenosis, and VSD	ccTGA, hypoplastic aortic arc, VSD, Ebstein anomaly of the tricuspid valve
Primary treatment	• Surgical APW, PAB, PDA ligation (with 1 month) • Combined Norwood-I and II operation with DKS anastomosis and plastic reconstruction of the pulmonary arteries (with 18 months) • Fontan procedure with lateral intra-atrial tunnel (with 4 years)	• Modified Norwood-I with RV-PA-Shunt (with 3 months) • Norwood-II procedure (with 5 months) • TCPC with 18 mm extracardial Goretex tunnel (with 27 months)	• Bidirectional Glenn anastomosis, severing of the pulmonary trunk and clipping of the azygos vein (with 4 months) • TCPC with 18 mm extracardial Goretex tunnel (with 22 months) • Interventional stenting of the Fontan tunnel due to kinking and thrombosis (with 23 months), re-stenting due to obstruction with protein-losing enteropathy (with 7 years)	• DKS anastomosis, 3.5 mm modified BT shunt and reconstruction of the aortic arc with pulmonary homograft patch (with 4 weeks of age) • Upper cavopulmonary anastomosis (bidirectional Glenn) with ligation of the mBT shunt (with 3.5 months) • TCPC with 16 mm extracardial Goretext tunnel (with 2.5 years)
Indication for cardiac catheterization	Ascites, edema of the lower extremities, pathological elastography	Chronic recurring abdominal pain, hepatosplenomegaly	Hepatomegaly, recurring abdominal pain, chronic diarrhea	Liver congestion with pathological elastography, protein-losing enteropathy in the past
TJLB	10 F sheath right jugular vein, three hepatic specimens from segment 5 through transjugular liver biopsy system	10 F sheath right jugular vein, three hepatic specimens from segment 5 through transjugular liver biopsy system	10 F sheath right jugular vein, three hepatic specimens from segment 5 through transjugular liver biopsy system	10 F sheath right jugular vein, three hepatic specimens from segment 5 through transjugular liver biopsy system
Time (min)	10	15	10	10
Complications	None	None	None	None
Histopathology	Moderate to severe fibrosis with periportal septa and fatty degeneration of 5% of the hepatocytes—Ishak score 4–5	Moderate fibrosis with periportal and porto-portal septa but intact architecture—Ishak score 3–4	Mild to moderate fibrosis with periportal and porto-portal septa but intact architecture—Ishak score 2–3	Mild fibrosis with periportal and porto-portal septa but intact architecture—Ishak score 2

Double ventricle anatomies	Case E	Case F	Case G
Sex	Female	Male	Male
Age	55 years	12 years	14 years
Primary diagnosis	Ebstein anomaly	Tetralogy of Fallot, LSVC	Diffuse intrapulmonary shunts, cirrhosis of the liver, hepatopulmonary syndrome
Primary treatment	• None	• Corrective heart surgery with 3 years of age • Stent-angioplasty (Valeo 6/20 mm) of the superior vena cava due to stenosis with 9 years	• None
Indication for cardiac catheterization	Ascites, edema of the lower extremities, right heart decompensation, pathological elastography	Ascites, hypoalbuminemia, suspicion of right heart decompensation	Assessment with possible occlusion of the intrapulmonary shunts due to massive desaturation, assessment of the cirrhosis
TJLB	10 F sheathe right jugular vein, three hepatic specimens from segment 5 through transjugular liver biopsy system	10 F sheath right jugular vein, three hepatic specimens from segment 5 through transjugular liver biopsy system	10 F sheath right jugular vein, three hepatic specimens from segment 5 through transjugular liver biopsy system
Time (min)	10	10	20
Complications	None	None	None
Histopathology	Moderate fibrosis with periportal and porto-portal septa but intact architecture—Ishak score 3–4	Fatty degeneration of 20% of the hepatocytes, moderate fibrosis with periportal and porto-portal septa but intact architecture—Ishak score 3–4	Pericentral lymphocytic and eosinophil infiltration, moderate fibrosis with periportal and porto-portal septa but intact architecture—Ishak score 3–4

HLHS, hypoplastic left heart syndrome; DILV, double inlet left ventricle; VSD, ventricular septal defect; APW, aortopulmonary window; PAB, pulmonary arterial banding; PDA, patent ductus arteriosus; DKS, Damus–Kaye–Stansel; RV, right ventricle; PA, pulmonary artery; TCPC, total cavopulmonary connection; TJLB, transjugular liver biopsy; ccTGA, congenital corrected transposition of the great arteries; mBT, modiefied Blalock-Taussig; LSVC, left superior vena cava.

Initially, a hemodynamic assessment was performed in room air (FiO_2_ 21%) before angiography and included measurements of aortic and ventricular pressure, superior and inferior vena cava pressure, Fontan tunnel pressures, pulmonary artery pressure, and pulmonary capillary wedge pressure. Angiography was performed in each case to identify possible Fontan pathway stenosis, venovenous collaterals, stenosis of the pulmonary arteries, or arterial–pulmonary collaterals.

In general, additional procedures, such as interventional closure of collaterals, dilatation and/or stenting of stenotic Fontan pathways, or endomyocardial biopsy, are performed whenever indicated after the initial hemodynamic assessment.

### 2.1. Technical aspects

**Figure 1:**
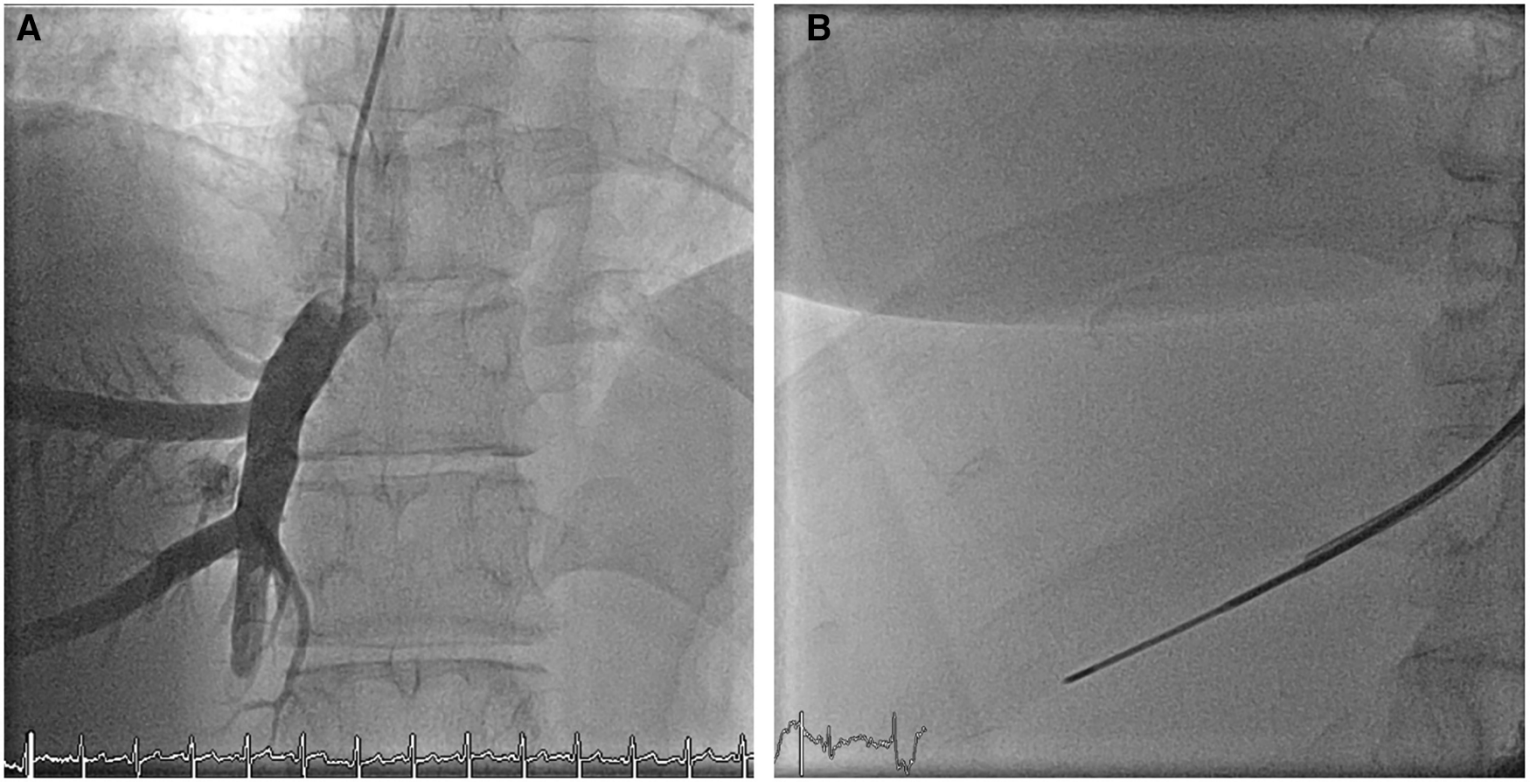
Fluoroscopy (**A**) mapping of the venous liver anatomy via an angiography (**B**) biopsy needle inserted over the stiff wire through the long sheath and positioned with its tip about 1.5 cm distal to the vein origin, liver segment V.

**Figure 2:**
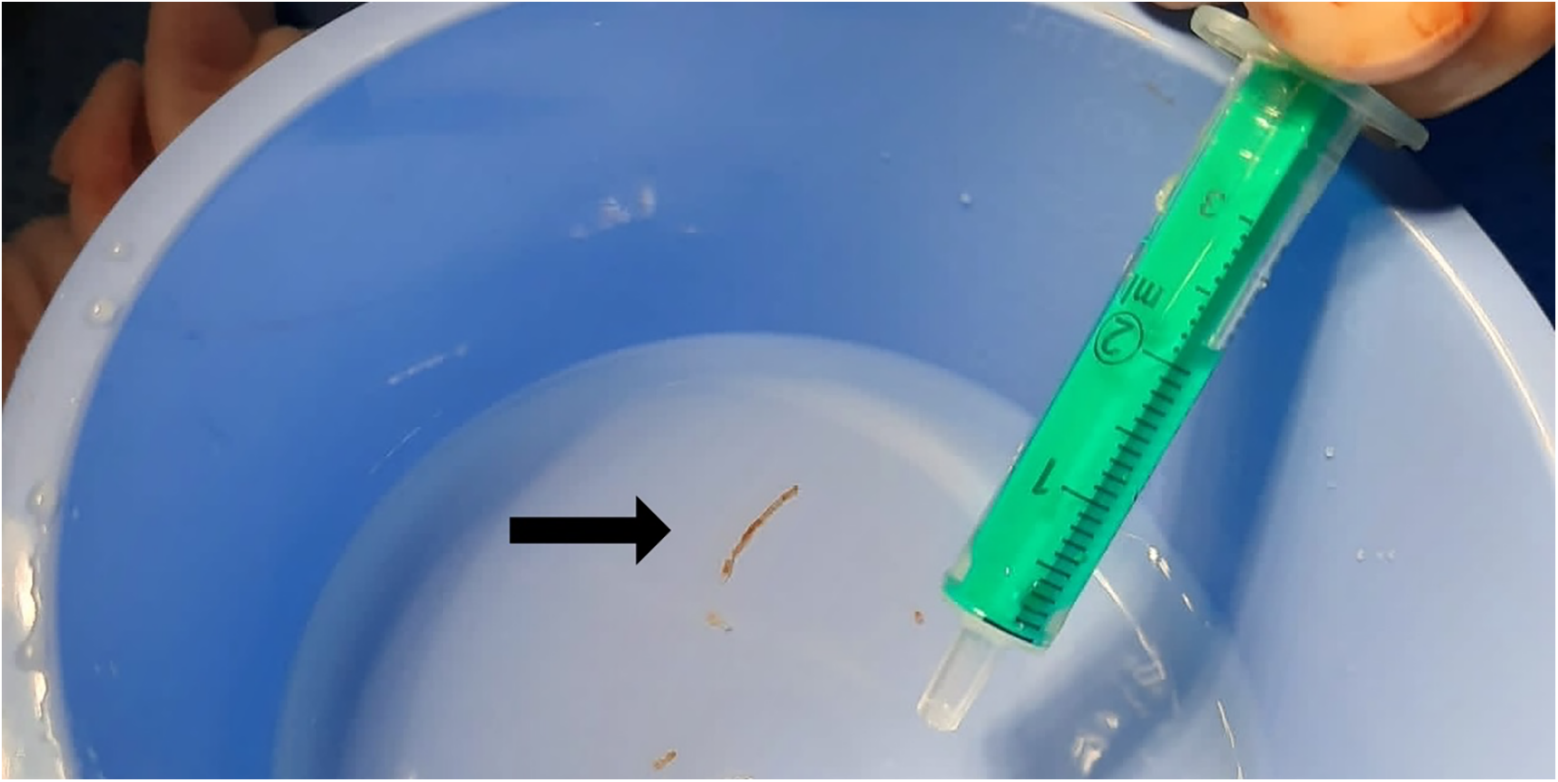
Obtained liver specimen.

Subsequently, jugular access was obtained in all patients at the site of liver localization using a 5 F Cook sheath, which was then exchanged for an 8 F sheath. A 5 F multipurpose catheter was inserted into the liver veins under fluoroscopic guidance. After performing hand angiography to delineate the local hepatic venous anatomy, an extra stiff x-change wire (Amplatz Super Stiff; Boston Scientific, Boston, MA, USA) was used to place the 10 F long sheath using a curved metal stiffener (Cook Flexor® Check-Flo®). The bioptome (Cook Medical Coaxial Biopsy Needle Set Quick-Core® 18 G) was inserted over the stiff wire through the long sheath and positioned with its tip about 1.5 cm distal to the vein origin, presenting enough thickness of the hepatic parenchyma to reduce the risk of capsular perforation. The 18 G biopsy needle was then maneuvered into the hepatic vein. Under fluoroscopy, the curved metal stiffener was turned anteriorly and inserted preferably into the right hepatic vein. The biopsy needle was then pushed through the vein wall into the hepatic parenchyma with a short thrust. The biopsy needle has a 2 cm throw and a 1.7 cm specimen notch. Three biopsies were obtained from either hepatic segment 5 or segment 8 (see Figures 1, 2). After completing the biopsies, the bioptome and sheath were retracted, and manual pressure was applied to the puncture site. A sonographic examination of the liver was performed within 10 min in the Cath lab to exclude hepatic perforation or subcapsular hematoma. There were no complications, especially perforation or bleeding. The technical success rate was 100%, with all obtained samples appropriate for histopathological diagnostics. The total additional procedure time was less than 20 min.

## 3. Histopathological examination

In all cases, the fibrosis was graded using standard scoring systems, including the congestive hepatic fibrosis score (CHS) (stages 0–4) and the modified Ishak congestive hepatic fibrosis (ICHF) score.

## 4. Discussion

FALD, one of the most common late-onset complications of Fontan palliation, carries an unfavorable prognosis (5). Medical centers that care for Fontan patients evaluate liver function using scoring systems and routine laboratory parameters, and they determine liver stiffness through a hepatic ultrasound or MRE. While laboratory parameters tend to indicate liver failure in later stages, elastography (ultrasound/MRI) correlates with liver fibrosis but relies on the expertise of the examiner and can be influenced by factors such as abdominal air or ascites. A liver biopsy remains the gold standard for assessing FALD and the extent of hepatopathy (6–8).

TJLB is typically performed as a preferred alternative when PLB is contraindicated because of factors such as thrombocytopenia, coagulopathy, or ascites. Various studies comparing PLB and TJLB have demonstrated no significant difference in terms of technical success and complication rates. These facts influenced us to introduce TJLB in our routine surveillance program for patients with suspected liver fibrosis and heart disease. Although specimen sizes obtained through TJLB are generally smaller, the histopathological findings are as accurate as those obtained through PLB (9–11).

Potential complications after a liver biopsy include capsular perforation of the liver, perforation of other abdominal organs such as the kidneys, and hemobilia (3, 12). PLB has been associated with cases of pneumothorax and abdominal wall aneurysm (13), while TJLB may involve technical complications related to the implantation of the sheath in the jugular vein (10).

Hemorrhage is the most commonly reported complication after liver biopsy, and it may be more pronounced in patients with impaired coagulation profiles and elevated central venous pressure, such as in Fontan patients. The incidence of hemorrhage varies between 4% and 18.5%, depending on the biopsy technique (traditional biopsy, ultrasound-guided PLB, or TJLB), the anatomy of the patient, and the experience of the operator (9, 13).

Studies conducted on groups of Fontan patients have shown a lower incidence rate of hemorrhage after TJLB (3.2%) compared with PLB (7.4%) (3, 12). The reduced hemorrhage risk associated with TJLB could be attributed to the possibility of precise anatomical mapping of the patient's circulation through angiography before the procedure. In addition, any potential bleeding in TJLB results in venous auto-transfusion, which is less concerning than an abdominal hemorrhage, especially in patients with reduced coagulation and elevated central venous pressure. Therefore, TJLB appears to be the preferred method in these patients to minimize the risk of hemorrhage. In all our initial patients presented here, we could not identify any of these complications confirming this well-known safety aspect.

The retrospective study by Borquez et al. (3) focused on 125 Fontan patients, with a median age of 17 years (ranging from 2 to 50.5 years), who underwent TJLB. The study reported a complication rate of 3.2% (four out of 125 cases), including two capsular perforations, one renal hematoma, and one case of hemobilia.

In the study conducted by Srinivasan et al. (12), which investigated 68 cases of ultrasound-guided PLB performed on 67 Fontan patients, with a median age of 20.2 years (ranging from 7.2 to 39 years), the only complication reported was hemorrhage in five out of 68 interventions, resulting in a hemorrhage incidence rate of 7.4%.

On the other hand, Govender et al. (13) evaluated the use of sonography-guided PLB in infants and children, including 470 patients who underwent 597 PLB procedures for various liver pathologies. The mean age of the patients was 10.5 years (ranging from 1 month to 21 years). In this study, 10 major complications were reported, representing a complication rate of 1.7%. These major complications included one case each of pneumothorax (0.2%) and abdominal wall pseudoaneurysm (0.2%), along with eight patients experiencing hemorrhage (1.3%). In addition, minor complications were noted in 49 patients (8.2%), with 35 cases (5.8%) of symptomatic subcapsular hematoma and 9 cases (1.5%) of small hemoperitoneum.

These studies provide valuable insights into the complication rates associated with TJLB and PLB in Fontan patients. TJLB appears to have a lower incidence of hemorrhage compared with PLB, making it a preferable approach in this patient population to reduce the risk of bleeding complications.

The study conducted by Meng et al. (9) involved comparing TJLB and PLB in 60 and 277 patients with various liver pathologies, respectively. In the TJLB group, the reported complications included one subcapsular hematoma, two cases of hematoma at the puncture site of the neck, and one case of paroxysmal supraventricular tachycardia. In contrast, the PLB group had 20 minor complications and four major complications. This study concludes that TJLB is a safe alternative to PLB.

Similarly, Sehmbhi et al. (10) performed a retrospective review of all liver biopsies conducted at a UK transplant center over 12 months. This study included 330 TJLB and 240 PLB procedures. The combined complication rate was 5.6%, with eight major complications (1.4%) and 24 minor complications (4.2%), and there was no significant difference between the two groups. However, the technical failure rate of TJLB was 4%, mainly because of difficulties in puncturing the internal jugular vein, failure to cannulate the hepatic vein, or an unstable sheath position.

In the comparison study by Hardman et al. (11), TJLB and image-guided PLB were performed in 36 and 202 cases, respectively. TJLB resulted in one major complication (2.8%) and two minor complications (5.5%), while PLB led to four major complications (1.9%) and 10 minor complications (4.9%). The authors concluded that both techniques showed similar efficacy and complication rates.

These studies collectively indicate that TJLB is a feasible and safe alternative to PLB in assessing liver pathology, especially in Fontan patients, and may offer advantages in terms of reduced risk of hemorrhage and overall complications. However, it is important to consider the specific clinical context and individual patient factors when deciding on the appropriate biopsy approach.

The modified ICHF score is considered a valuable tool for assessing liver biopsies in Fontan patients. Unlike other traditional histopathological scoring systems, the ICHF score is particularly suitable for evaluating the liver tissue in these patients.

In addition to the histopathological evaluation, investigations have shown that MRE is useful for the longitudinal follow-up of liver stiffness in Fontan patients. MRE allows non-invasive assessment of liver fibrosis by measuring liver stiffness, and it has been found to correlate significantly with the ICHF scoring and invasive hemodynamic parameters such as the mean pressure in the Fontan circulation and the cardiac index.

Therefore, when the MRE score is greater than 5 kPa, combining an invasive hemodynamic examination with a TJLB is recommended. This integrated approach can provide comprehensive information about the liver condition in Fontan patients and help manage their liver disease.

By using MRE and ICHF scoring in combination with TJLB, healthcare providers can better monitor the liver health of Fontan patients and make informed decisions regarding their treatment and long-term follow-up care (14).

Whenever there is a need for hemodynamic assessment in Fontan patients because of clinical or hemodynamic alterations during long-term follow-up (e.g., protein-losing enteropathy, reduced exercise performance, and increasing cyanosis), catheter investigations are commonly used to evaluate cardiac output and pulmonary pressures and calculate pulmonary vascular resistance. These investigations often require sedation or general anesthesia to ensure patient comfort and cooperation during the procedure.

Based on the literature reviewed and our initial results, we would support the management approach where TJLB can be easily incorporated into the catheter investigation procedure with minimal additional effort in these patients. The procedure itself is comparable to many other interventional measures; therefore, skilled cardiac interventionalists can safely perform the TJLB without significantly prolonging the overall procedure time. This approach streamlines patient management by reducing the number of separate investigations needed and, in turn, enhances patient comfort and convenience.

By combining hemodynamic assessment with TJLB, healthcare providers can obtain comprehensive information about cardiac and liver health in univentricular Fontan patients or patients with other biventricular circulation during a single session. This integrated approach offers several benefits, including simplifying the overall evaluation process, reducing the need for multiple interventions, and ultimately increasing patient comfort and satisfaction.

## 5. Limitations

Its relatively modest sample size inherently constrains this case series. Nonetheless, given the novelty of the introduced method within our management portfolio, we were able to establish its feasibility and safety.

## 6. Conclusion

TJLB is a valuable procedure that can be performed during routine hemodynamic evaluations in patients, with minimal additional time required. By conducting TJLB during routine cardiac catheterization and under conscious sedation, both hemodynamic data and liver biopsies can be obtained simultaneously, reducing the procedure time and overall count of interventions.

This alternative method of obtaining liver specimens for FALD care has shown potentially lower complication rates, such as bleeding and hematoma, compared with other techniques, despite the challenges posed by elevated central venous pressure and reduced coagulation profiles in Fontan patients. In addition, during the catheterization, common vascular anomalies in Fontan anatomy (e.g., heterotaxy) can be easily assessed through angiography, further minimizing the risk of complications.

Furthermore, in the event of a possible hemorrhage during the TJLB, venous auto-transfusion occurs without clinical consequences, adding to the safety of the procedure.

Considering the lower complication rates reported, our own initial results, and the ease of assessing vascular anomalies, we recommend incorporating cardiac catheterization with simultaneous TJLB as a standard approach in the routine surveillance program for all patients with Fontan palliation, especially when clinical scores and/or liver elastography trends indicate a deterioration of liver function or worsening of FALD. This integrated approach ensures a comprehensive evaluation of both cardiac and liver health, promoting effective management and timely interventions when necessary.

## Data Availability

The original contributions presented in the study are included in the article/Supplementary Material, further inquiries can be directed to the corresponding author.
